# A Comparison of Three Methods of Height Estimation and Their Impact on Low Tidal Volume Ventilation in a Mixed Ethnicity Intensive Care Unit: A Real-World Experience

**DOI:** 10.7759/cureus.9221

**Published:** 2020-07-16

**Authors:** Keevan Singh, Natalia Gocool

**Affiliations:** 1 Anaesthesia and Intensive Care, University of West Indies, San Fernando, TTO; 2 Anaesthesia and Intensive Care, San Fernando General Hospital, San Fernando, TTO

**Keywords:** ventilation, low tidal volume ventilation, ards, predicted body weight, acute lung injury

## Abstract

Background

Height measurement is crucial for calculating predicted body weight (PBW) and establishing low tidal volume ventilation (LTVV). However, standing height is usually unavailable in critically ill patients and supine height may be difficult to obtain.

Objective

We investigated whether there were any significant differences in tidal volumes (VT) obtained using PBW derived from supine, forearm, and lower leg lengths in an intensive care unit (ICU) setting.

Methods

Supine, forearm and lower leg lengths were measured in 100 mechanically ventilated patients. Limb lengths were converted to height and PBW calculated using published formulae. The 6 mL/kg VT for the supine (sVT), forearm (fVT), and lower leg (lVT) methods were compared to each other and to visually estimated VT (estVT).

Results

Forearm length produced the greatest height estimate, leading to a significantly greater tidal volume fVT (437.6 ± 62.1 mL) compared with sVT (385.5 ± 63.8 mL) and lVT (369.1 ± 66.4 mL), (p < .001). There was no significant difference between lVT and sVT, (p = .169). On Bland Altman analysis, the lowest bias was found between lVT and sVT (−16.4 ± 36.0 mL, 95% limits of agreement (LOA) [−86.9, 54.1]), whereas fVT had a bias of 52.1 ± 41.5 mL, 95% LOA [−29.1, 133.4] compared to sVT. The fVT was significantly greater than sVT and lVT in all sexes and ethnic groups (p < .05).

Conclusion

Lower leg length may be a suitable alternative to supine height to facilitate the application of LTVV in an ICU setting.

## Introduction

Mechanical ventilation carries the risk of precipitating and exacerbating pre-existing lung injury [1]. The pathophysiological mechanisms involved in ventilator-associated lung injury (VALI) include alveolar-capillary barrier disruption and inflammation secondary to volutrauma, barotrauma, atelectrauma and biotrauma. The traditional use of high tidal volumes (VTs) between 10 to 15 mL/kg predicted body weight (PBW) has been shown to be a critical mediator of VALI [2-4], which is associated with increased morbidity and mortality [5].

A key component of a lung-protective ventilatory strategy, therefore, involves the use of low tidal volume ventilation (LTVV) between 4 to 8 mL/kg PBW [6-9]. Since the publication of the Acute Respiratory Distress Syndrome Clinical Network (ARDSNet) trial, which demonstrated a mortality benefit with the use of VTs 6 mL/kg versus 12 mL/kg PBW, LTVV has become a recommended standard of care for patients with ARDS [7]. There is also mounting evidence to support using low VTs in non-ARDS patients [9,10].

However, LTVV remains underutilized [11,12], with one possible reason being that PBW is not used to set VT. The importance of using PBW is based on the fact that lung volume is directly related to height [13-15]. However, in some ICUs VT is based on visual estimates of height or weight, often leading to inappropriately high VTs [16-21].

There is no consensus on the most suitable method of height measurement in the critically ill. The gold standard is standing height, which is often impossible to obtain [22]. Supine height, the most common alternative, may also be prone to various errors. These may include a tendency to measure the contours of the patient, difficulty due to body distortion, the inability of critically ill patients to lie completely supine, attachment of tubes, lines, and lack of patient cooperation.

The lack of a simple method for height estimation can pose a major barrier to the application of LTVV. The forearm length, arm span, demi-span, tibia length, and lower leg length have all been examined [22]. Since previous work has shown that measurements of forearm length and lower leg length correlate best with standing height [23-25], we chose to investigate these two methods for PBW calculation and VT setting in our ICU.

This study aimed to investigate whether there were any significant differences in the calculated 6 mL/kg VT when using PBW derived from supine height and height estimated from forearm and lower leg lengths in the ICU. Secondary objectives included comparisons with the clinician’s visual 6 to 8 mL/kg VT estimate (estVT), analysis of the trends in VT between different sexes and ethnicities, and assessment of inter-observer variability in the measurements.

## Materials and methods

This prospective observational study was conducted during the period March to July 2017 in the mixed medical-surgical ICU of a tertiary teaching hospital.

Prior approval was obtained from the representative ethics committee of the hospital. Written informed consent was waived by the bio-ethics committee due to the observational and non-invasive nature of the study.

To estimate a 50% prevalence of inaccurate height measurement by any method, for a 10% precision and 95% level of confidence, a sample size of 100 patients was estimated using Epi Info version 7.0 (CDC, Atlanta, GA).

Patients 18 years and older requiring intubation and mechanical ventilation using volume assist control or volume synchronized intermittent mandatory ventilation modes were included in the study. Patients were excluded if they were unable to lie completely supine or if their left upper and lower limbs were not amenable to measurement (due to dressings, deformity, or amputation).

Study measurements

Three methods of height estimation were investigated (Table 1).

**Table 1:** Table showing the three different measurement methods used for height estimation.

Method	Description
Supine Length	Vertex of head to heel with patient supine
Forearm Length [23]	Olecranon to mid-point of styloid process with left forearm bent across chest and fingers pointing to contralateral shoulder
Lower Leg Length (simplified Chumlea method) [25]	Upper border of patella to heel with left leg supine

Measurements were performed independently by two registered ICU nurses. All measurements were taken to the nearest 0.5 cm using a soft 150 cm tape.

The British Association for Parenteral and Enteral Nutrition (BAPEN) formulae were utilized for converting limb lengths to height, as these produced favorable results in previous studies investigating the use of forearm and lower leg length for height estimation and VT setting [23-27].

After the conversion of limb length to height, PBW was derived as per ARDSNet formula and then used to calculate the appropriate 6 mls/kg VT [7]. Estimated tidal volume (estVT) was recorded from the patient notes and represents the clinician's visual estimate of the appropriate VT. This measurement, estVT, would also have been the input VT on the ventilator. No actual ventilator derived measurements were used in the study. 

Data collection

Patients admitted to the ICU during the specified period were screened for entry into the study. Patients not meeting the eligibility criteria were replaced by the next eligible subject until the appropriate sample size was obtained. Information regarding each patient’s age, gender, ethnicity, admitting diagnosis, initial mode of ventilation, and estVT was documented by the investigator using a manual Data Collection Instrument.

**Figure 1:**
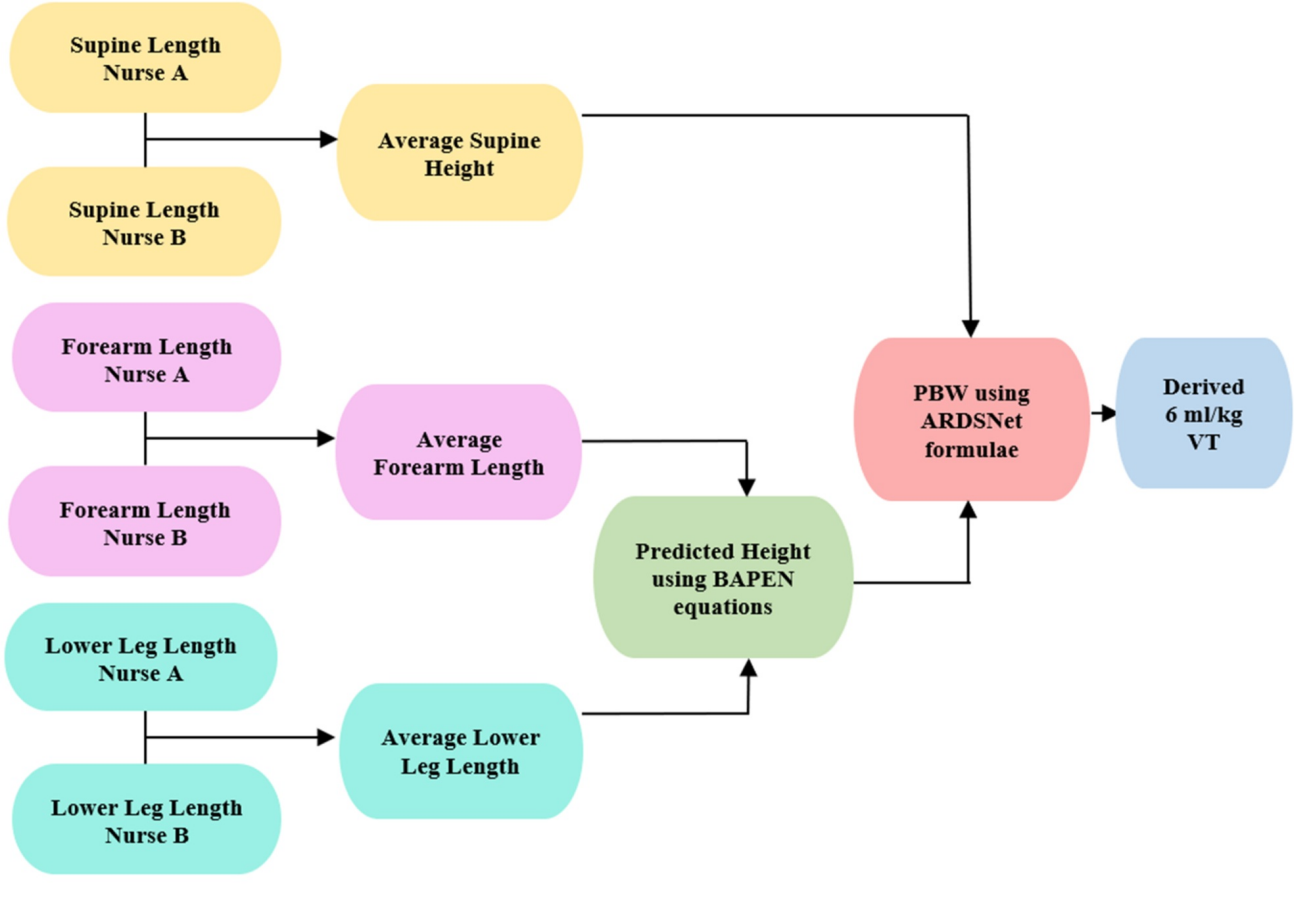
Flow chart showing the steps from data collection to derived tidal volumes.

Two ICU nurses, on duty on the day of initiation of mechanical ventilation, carried out the three measurements independently of each other. In addition to a verbal explanation of the procedure, instructions on the measurement techniques were included on the data collection form issued to each nurse. Figure 1 summarises the process from data collection to VT calculation [26-28]. 

Statistical analysis

Data were analyzed using the IBM Statistical Package for the Social Sciences; version 22.0 (SPSS Inc., Chicago, IL). One-way analysis of variance (ANOVA) was used to assess for any statistically significant differences in the VTs obtained using the three different measurements, to compare the three methods with estVT and to assess sex- and ethnicity-based differences among the VTs. Multiple comparisons between the individual VTs were done post-hoc using Tukey's honestly significant difference (HSD) test. The bland-Altman analysis was used to determine the bias and LOA between the different methods. The intraclass correlation coefficient (ICC) was used to assess inter-observer agreement between the nurses in the three measurements.

## Results

One hundred patients (58 males and 42 females) were enrolled from a total of 156 admissions during the study period. Most patients were of Indian descent (69%), with the remainder being of African (28%) or mixed (3%) descent. Of the patients studied 66% were surgical admissions and 34% medical. 

Forearm derived VT (fVT) was the largest of all the derived VTs (fVT, 437.6 ± 62.1 mL), whilst lower leg derived VT (lVT) was the smallest (lVT, 369.1 ± 66.4 mL). This fVT was significantly different from lVT, sVT and estVT (p < .001).

On post-hoc testing using Tukey's HSD test, similar results were obtained in the subgroup analyses based on gender and the two major ethnic groups, with the fVT being statistically significantly greater than the VT derived using supine or lower leg lengths in all subgroups (p < .05). In all subgroups, the lVT was not significantly different from the sVT (p = .169) (Table 2),

**Table 2:** Table showing the mean ± SD height, PBW and VT using the different measurement methods and the estVT in the study population and subgroups. * p < .001 for fVT vs lVT and fVT vs sVT, † p < .05 for estVT vs fVT and estVT vs lVT. PBW, predicted body weight; VT, tidal volume; estVT, visually estimated tidal volume; fVT, forearm derived tidal volume; lVT, lower leg derived tidal volume; sVT, supine derived tidal volume.

Method		Supine	Forearm	Lower Leg	estVT
Total Sample	Height (cm)	170.2 ± 10.1	179.9 ± 9.7	167.2 ± 10.5	-
	PBW (kg)	64.3 ± 10.6	72.9 ± 10.3	61.5 ± 11.0	-
	VT (ml)	385.5 ± 63.8	437.6 ± 62.1*	369.1 ± 66.4	397.4 ± 56.4 †
Males	Height (cm)	174.9 ± 7.8	185.4 ± 7.9	172.9 ± 7.2	-
	PBW (kg)	70.4 ± 7.0	79.8 ± 7.2	68.5 ± 6.6	-
	VT (ml)	422.3 ± 41.7	478.7 ± 43.1*	411.4 ± 40.1	418.3 ± 51.3
Females	Height (cm)	163.6 ± 9.5	172.2 ± 6.1	159.3 ± 9.1	-
	PBW (kg)	55.8 ± 8.9	63.5 ± 5.4	51.80 ± 8.1	-
	VT (ml)	334.7 ± 53.4	380.9 ± 32.2*	310.8 ± 48.9	368.5 ± 50.5
African	Height (cm)	174.5 ± 9.2	182.0 ± 9.6	170.8 ± 10.0	-
	PBW (kg)	68.1 ± 9.4	74.6 ± 10.2	64.7 ± 10.4	-
	VT (ml)	408.7 ± 56.4	447.8 ± 61.5	388.7 ± 63.4	413.6 ± 57.7
Indian	Height (cm)	168.5 ± 10.1	179.1 ± 9.8	165.8 ± 10.6	
	PBW (kg)	62.7 ± 10.9	72.4 ± 10.6	60.3 ± 11.3	
	VT (ml)	376.4 ± 65.6	434.5 ± 63.4*	362.0 ± 67.6	392.4 ± 55.7

In males, forearm length led to higher VTs (60 mL) on average compared to physician estimated VT (p < .001). In females, however, estVT was not statistically different from VT derived using the forearm measurement (p = .621), which was generally the largest of the three measurements. In patients of Indian descent, significant differences existed between the estVT and VT derived from forearm length (p = .001) and lower leg length (p = .026).

Bland Altman analysis

**Figure 2:**
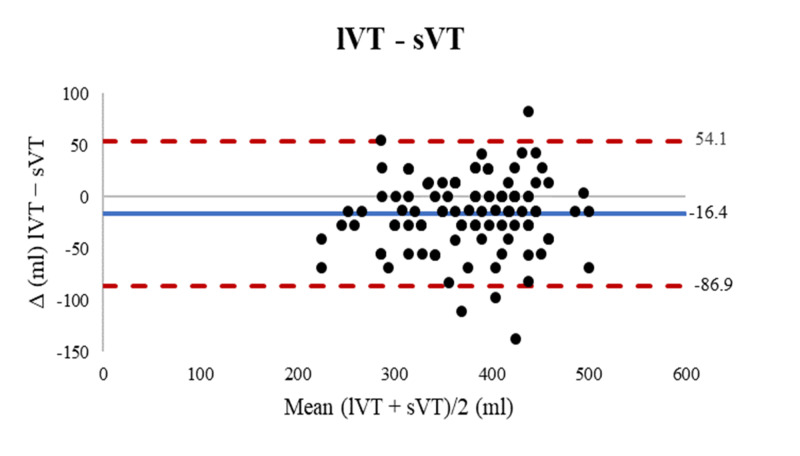
Bland Altman plots comparing lVT and sVT. Blue solid line represents the bias and red dashed lines represents the limits of agreement. lVT: lower limb derived tidal volume, sVT: supine derived tidal volume.

Compared with the supine method, the bias with the lower leg method (lVT) was −16.4 ± 36.0 mL, 95% LOA [−86.9, 54.1] (Figure 2).

**Figure 3:**
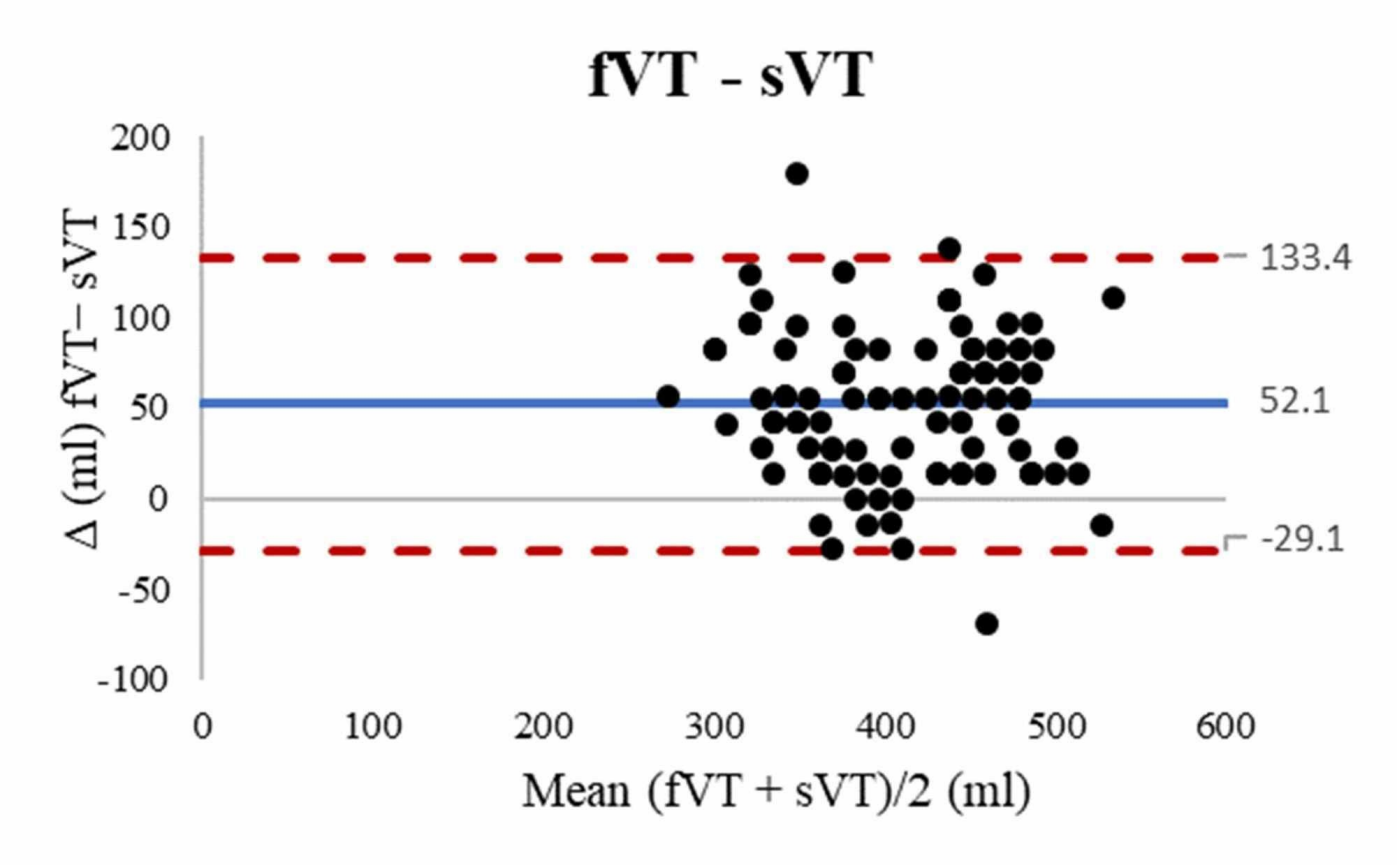
Bland Altman plots comparing fVT and sVT. Blue solid line represents the bias and red dashed lines represent the limits of agreement. fVT: forearm derived tidal volume, sVT: supine derived tidal volume.

**Figure 4:**
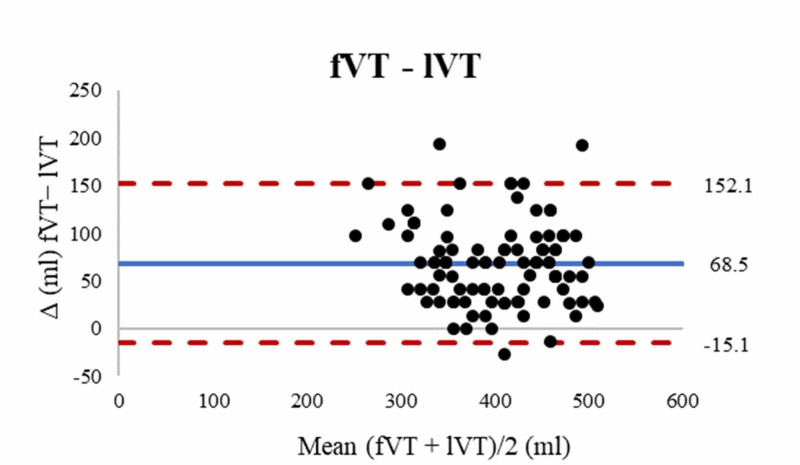
Bland Altman plot fVT and lVT. Blue solid line represents the bias and red dashed lines represent the limits of agreement. fVT: forearm derived tidal volume, lVT: lower leg derived tidal volume.

In contrast, the forearm method tended to overestimate VT compared with the supine method (bias 52.1 ± 41.5 mL, 95% LOA [−29.1 to 133.4]), as seen in Figure 3, and the lower leg method (bias 68.5 ± 42.6 mL, 95% LOA [−15.1 to 152.1]) (Figure 4). 

Inter-observer variability

The ICC also demonstrated good inter-observer agreement in the forearm measurements (ICC .881, 95% CI [.824, .920], p < .001). Excellent inter-observer agreement was shown for both the supine measurements (ICC .952, 95% CI [.929, .968], p < .001) and the lower leg measurements (ICC .973, 95% CI [.960, .982], p < .001) [29].

## Discussion

Previous research has shown that the use of both the forearm and lower leg lengths for height estimation tends to reduce the calculated PBW and decrease the likelihood of administering high VTs [23-25]. However, in our study, this was true only for the lower leg length when compared with supine height.

Studies in various populations have shown significant correlations between lower leg length and standing height [22-25]. Our investigation demonstrated that VT derived from lower leg length was not statistically different from VT derived from supine height, despite providing lower values, on average 16 mL, across all sexes and ethnicities.

The VT derived using lower leg length correlated well with the VT derived from standing height in a study by Bojmehrani and colleagues, with only 15% of patients having VT errors over 10% with the lower leg method. The lower leg method was also found to be at least as efficient as the supine method, however, maximum errors were lower when using lower leg length. The use of height from lower leg length also led to the smallest errors in the calculation of PBW and VT compared with visual estimation or the use of forearm length [24]. These findings are consistent with our results, which showed that the lower leg length provided the lowest VT estimates.

Numerous studies have confirmed a significant correlation between forearm length and standing height, and various population-specific formulae have been developed to describe this relationship using regression analysis [22]. The British Association for Parenteral and Enteral Nutrition (BAPEN) equations used in Caucasian populations provided similar estimates to standing height, with little impact on derived VT [23,24]. In our study, however, the height derived from forearm length was significantly greater than the supine height, with a consequently greater estimated VT (MD (mean difference) 52 mL, p < .001). This suggests the need for further investigation into the relationship between forearm length and height and the development of formulae specific to our local population.

It was noteworthy that there was a smaller difference between the fVT and the sVT in patients of African descent compared with those of Indian descent (38.1 mL, p = .047 and 58.1 mL, p < .001 respectively). The BAPEN equations for converting forearm length to height may, therefore, be suitable in the African subgroup, but less so in the Indian population. However, investigation using a larger patient cohort is needed for confirmation. 

Our study demonstrated that the estVT in females was significantly greater than the sVT (MD 33.7 mL, p < .01) and the lVT (MD 57.7 mL, p < .001), particularly in females of Indian descent. However, the estVT was not significantly different from the fVT, which tended to overestimate VT in the general study population. Since a value for height was unavailable at the onset of mechanical ventilation, this high estVT may indicate clinicians’ tendency to overestimate height and weight for female patients. This finding is consistent with international studies which revealed that women, as well as short and obese patients, are less likely to be provided with protective ventilation [20,24,30]. 

Post hoc testing showed that in our population the fVT was statistically significantly greater than the sVT or lVT (MD 52.1 mL, p < .001 and 68.5 mL, p < .001 respectively). However, it is difficult to ascertain whether these mean differences in VT would be clinically significant. Considering that the average PBW of the study population, by all three methods, was 66 kg, the forearm length would produce a mean 6.6 mL/kg VT versus a 5.8 mL/kg and 5.5 mL/kg VT from supine and lower leg length, respectively (Table 2). Although these values lie within the range for protective ventilation, the Bland-Altman analysis showed that forearm length overestimated VT by up to 133 mL (2.0 mL/kg) and 152 mL (2.3 mL/kg) compared with the supine and lower leg length respectively (Figures 3-4). In contrast, the lower leg length had the lowest bias (− 16.4 mL) and overestimated VT by up to only 54 mL (0.8 mL/kg) compared with the supine method (Figure 2). Thus, the use of the lower leg length as an alternative to supine height may offer the best safety margin in VT setting in our population.

The lower leg method for height estimation is advantageous compared to other methods due to the ease of a single measurement by one operator, the lack of requirement to lie the patient completely supine, and the minimal risk of dislodgement of invasive lines, which may be commonly placed on the upper limbs. The best inter-observer agreement was also noted with this method (ICC .973, p < .001).

A major limitation of this study was the inability to validate the calculated VT from the three methods against the gold standard VT from standing height. However, the supine height is usually the only available reference in many ICUs.

The use of the specific BAPEN equations may be another contributory factor to the differences observed in the derived body weights. It is possible that another equation for the conversion of forearm length to height would have been better suited to our specific ethnic makeup. 

## Conclusions

Where the gold standard is unavailable, height derived from lower leg length may be used as an alternative to supine height for setting VT especially when LTVV is desired. While height derived from lower leg length approximated supine height in the multi-ethnic population studied, other anthropometric measures can overestimate height.
